# Area deprivation and demographic factors associated with diabetes technology use in adults with type 1 diabetes in Germany

**DOI:** 10.3389/fendo.2023.1191138

**Published:** 2023-08-01

**Authors:** Marie Auzanneau, Alexander J. Eckert, Sebastian M. Meyhöfer, Martin Heni, Anton Gillessen, Lars Schwettmann, Peter M. Jehle, Michael Hummel, Reinhard W. Holl

**Affiliations:** ^1^ Institute of Epidemiology and Medical Biometry, Ulm University, Ulm, Germany; ^2^ German Center for Diabetes Research (DZD), Munich-Neuherberg, Germany; ^3^ Institute for Endocrinology and Diabetes, University of Lübeck, Lübeck, Germany; ^4^ Division of Endocrinology and Diabetology, Department of Internal Medicine 1, University Hospital Ulm, Ulm, Germany; ^5^ Department for Diagnostic Laboratory Medicine, Institute for Clinical Chemistry and Pathobiochemistry, University Hospital Tübingen, Tübingen, Germany; ^6^ Department of Internal Medicine, Sacred Heart Hospital, Muenster, Germany; ^7^ Division of Health Economics, Department of Health Services Research, Carl von Ossietzky University of Oldenburg, Oldenburg, Germany; ^8^ Department of Internal Medicine I, University Medicine, Academic Hospital Paul-Gerhardt-Stift, Martin-Luther-University Halle-Wittenberg, Lutherstadt Wittenberg, Germany; ^9^ Research Group Diabetes e.V., Helmholtz Center Munich, Munich-Neuherberg, Germany

**Keywords:** Type 1 diabetes, adults, diabetes technology, CGM, pump, age, gender, deprivation

## Abstract

**Introduction:**

Diabetes technology improves glycemic control and quality of life for many people with type 1 diabetes (T1D). However, inequalities in access to diabetes technology exist in many countries. In Germany, disparities in technology use have been described in pediatric T1D, but no data for adults are available so far. We therefore aimed to analyze whether demographic factors and area deprivation are associated with technology use in a representative population of adults with T1D.

**Materials and methods:**

In adults with T1D from the German prospective diabetes follow-up registry (DPV), we analyzed the use of continuous subcutaneous insulin infusion (CSII), continuous glucose monitoring (CGM), and sensor augmented pump therapy (SAP, with and without automated insulin delivery) in 2019-2021 by age group, gender, migration background, and area deprivation using multiple adjusted regression models. Area deprivation, defined as a relative lack of area-based resources, was measured by quintiles of the German index of Multiple Deprivation (GIMD 2015, from Q1, least deprived, to Q5, most deprived districts).

**Results:**

Among 13,351 adults with T1D, the use of technology decreased significantly with older age: CSII use fell from 56.1% in the 18−<25-year age group to 3.1% in the ≥80-year age group, CGM use from 75.3% to 28.2%, and SAP use from 45.1% to 1.5% (all p for trend <0.001). The use of technology was also significantly higher in women than in men (CSII: 39.2% vs. 27.6%; CGM: 61.9% vs. 58.0%; SAP: 28.7% vs. 19.6%, all p <0.001), and in individuals without migration background than in those with migration background (CSII: 38.8% vs. 27.6%; CGM: 71.1% vs. 61.4%; SAP: 30.5% vs. 21.3%, all p <0.001). Associations with area deprivation were not linear: the use of each technology decreased only from Q2 to Q4.

**Discussion:**

Our real-world data provide evidence that higher age, male gender, and migration background are currently associated with lower use of diabetes technology in adults with T1D in Germany. Associations with area deprivation are more complex, probably due to correlations with other factors, like the higher proportion of migrants in less deprived areas or the federal structure of the German health care system.

## 1 Introduction

Over the past few years, considerable advances in diabetes technology have revolutionized the management of type 1 diabetes (T1D). Not only continuous glucose monitoring systems (CGM) and continuous subcutaneous insulin infusion (CSII or insulin pumps), but also innovative systems connecting both devices with algorithms to facilitate automated insulin delivery (AID, or “hybrid closed loop”, HCL) have been increasingly used by people with T1D in high-income countries over the past decade (1–3). Numerous studies indicate that the use of these different devices is associated with better glycemic control (2, 4–6), less severe hypoglycemia (2, 5, 6), and improved quality of life (6–8) in both children and adults with T1D. However, significant inequalities in use of modern diabetes technology have been reported in many countries. In pediatric populations, persistent or widening racial-ethnic and/or socioeconomic disparities in the use of CSII and CGM have been described in the US (3, 4, 9–11), in Canada (12), in New-Zealand (13), in the UK (4, 14), or in Germany (4, 9, 15). In adults, the use of diabetes technology is still less widespread than in children and only few studies were performed. Nevertheless, ethnic disparities in the use of CSII, CGM and also AID, have been described in the US (16, 17), as well as ethnic and socioeconomic disparities in CSII and CGM use in the UK (18).

The influence of demographic or socioeconomic factors on the use of diabetes technology in adults has not been analyzed to date in Germany. However, information on the actual use of the different diabetes treatment devices in the entire population, including underrepresented groups, such as migrants, the elderly, or the socioeconomically disadvantaged, is important. Studies focusing on disadvantaged populations point out that the use of CSII and CGM helps to reduce adverse events and to improve HbA1c levels in these groups and that diabetes technology has therefore the potential to reduce disparities in diabetes outcomes (19–21). Nevertheless, if those who could benefit most from technologies have less access to it, and if these disparities increase as diabetes technologies advance, disparities in diabetes outcomes are expected to worsen (22, 23). To properly assess this issue, it is necessary to know accurately the current utilization rates of commercially available diabetes treatment devices in different population subgroups. Therefore, we aimed to analyze recent technology use in Germany in a representative population of adults with T1D by age, gender, migration background, and area deprivation (as defined in the following section).

## 2 Materials and methods

### 2.1 Data source and study population

In this cross-sectional study, we used data from the multicenter, diabetes prospective follow-up registry (DPV). As of September 2022, the DPV registry comprised demographic and clinical data of about 705,000 patients with any type of diabetes, documented by 507 pediatric and adult health care facilities, of which 456 are located in Germany. All participating centers transmit twice a year the locally collected data in pseudonymized form to Ulm University, Germany. After plausibility checks and corrections, the Ulm University aggregates the data into an anonymized database for benchmarking and medical research. Data collection and analysis were both approved by the ethics committee of the Medical Faculty of Ulm University (Number 314/21) and by local review boards of the participating centers. In the present study, we included data documented between 2019 and 2021 of individuals diagnosed with T1D since at least three months, aged ≥18 years, with residence in Germany. T1D was identified by a clinical diagnosis at the age of at least 6 months and the documentation of insulin use.

### 2.2 Demographic variables and area deprivation

Age was divided into the following groups: 18-<25 year, 25-<40 years, 40-<60 years, 60-<80 years and ≥ 80 years. Migration background was defined as place of birth outside Germany for the patient or at least for one of his parents. Area deprivation was assessed using the German Index of Multiple Deprivation of the year 2015 (GIMD 2015). The concept of area deprivation can be defined as a lack of area-based resources, compared to the society in which one lives (24, 25). As described in previous publications (24, 26), the GIMD encompassed aggregated data at district level in seven deprivation domains differently weighted: income (25%), occupation (25%), education (15%), municipal/district revenue (15%), social capital (10%), environment (5%), and security (5%). Districts were categorized into area deprivation quintiles from Q1 (lowest deprivation quintile) to Q5 (highest deprivation quintile). We used individual postal code of patient’s residences to assign them to districts and consequently to GIMD quintiles.

### 2.3 Use of diabetes technology

We investigated any use of insulin pump/continuous subcutaneous insulin infusion (CSII), sensor/continuous glucose monitoring (CGM), and sensor augmented pump therapy (SAP) in the observation period. SAP use was defined as simultaneous use of insulin pump and sensor, connected or not with algorithms for automated insulin delivery (AID).

### 2.4 Statistical analysis

Data documented between 2019 and 2021 were aggregated per individual as maximum (technology use documented once or not during this period) or median (other variables). Using multiple logistic regressions, we analyzed the proportion of individuals using CSII, CGM, and SAP by gender, age group, migration background, and area deprivation. All models were adjusted for diabetes duration group (0-<5 years, 5-<10 years, 10-<20 years, and ≥20 years), and when possible for gender and age groups (see above). Multiple regressions models including all factors together (gender, age group, migration background, and area deprivation) were additionally performed as sensitivity analysis. In addition, interactions between migration background and area deprivation were analyzed. Associations of technology use (CSII, CGM, and SAP) with HbA1c were analyzed using multiple linear regressions in each gender, age, migration, and deprivation subgroup (stratification). All models were adjusted for diabetes duration group, and when possible for gender and age groups.

Results of regression analyses are presented as coefficients and as adjusted proportions (least square means) with 95%-confidence intervals (95%-CI). Descriptive data are given as median with lower and upper quartiles for continuous variables and as percentage for binary variables. A p-value <0.01 in two-sided tests was considered statistically significant. Statistical analyses were conducted using SAS version 9.4 (build TS1M7, SAS Institute Inc., Cary, NC).

## 3 Results

The study population comprised 13,351 adults with T1D, with median age of 30.9 years [lower−upper quartile: 19.0−55.8 years] and median diabetes duration of 13.4 years [7.2−23.8 years] (**Table 1**). Overall, 36.4% used a CSII, 59.0% at least once a CGM (37.8% at least 90 days per year), and 27.1% both devices (22.6% SAP without AID and 4.5% SAP with AID).

**Table 1:** Characteristics of the study population.

	Median (lower-upper quartile)	n, percent (%)
Age, years	30.9 (19.0−55.8)	
Age groups		
18 - <25 years		5,902 (44.2)
25 - <40 years		1,915 (14.3)
40 - <60 years		2,906 (21.8)
60 - <80 years		2,160 (16.2)
≥ 80 years		468 (3.5)
Sex		
Male		7,132 (53.4)
Female		6,219 (46.6)
Migration background*		
Without Migration background		4,015 (30.1)
With Migration background		1,275 (9.5)
Not documented		8,061 (60.4)
Diabetes duration, years	13.4 (7.2−23.8)	
BMI **	25.7 (24.8–22.1)	
HbA1c, %	7.65 (6.88–8.69)	
Use of CSII		4,860 (36.4)
Use of CGM		
Any use		7,877 (59.0)
Use ≥ 90 days/year		5,047 (37.8)
Use of SAP		
All SAP		3,618 (27.1)
Only AID		601 (4.5)
All patients		13,351 (100.0)

Unadjusted data. *defined as birth of the patient himself or at least one of his parents outside of Germany.**Body Mass Index (kg/m^2^).

### 3.1 Technology use by age group

**Figure 1:**
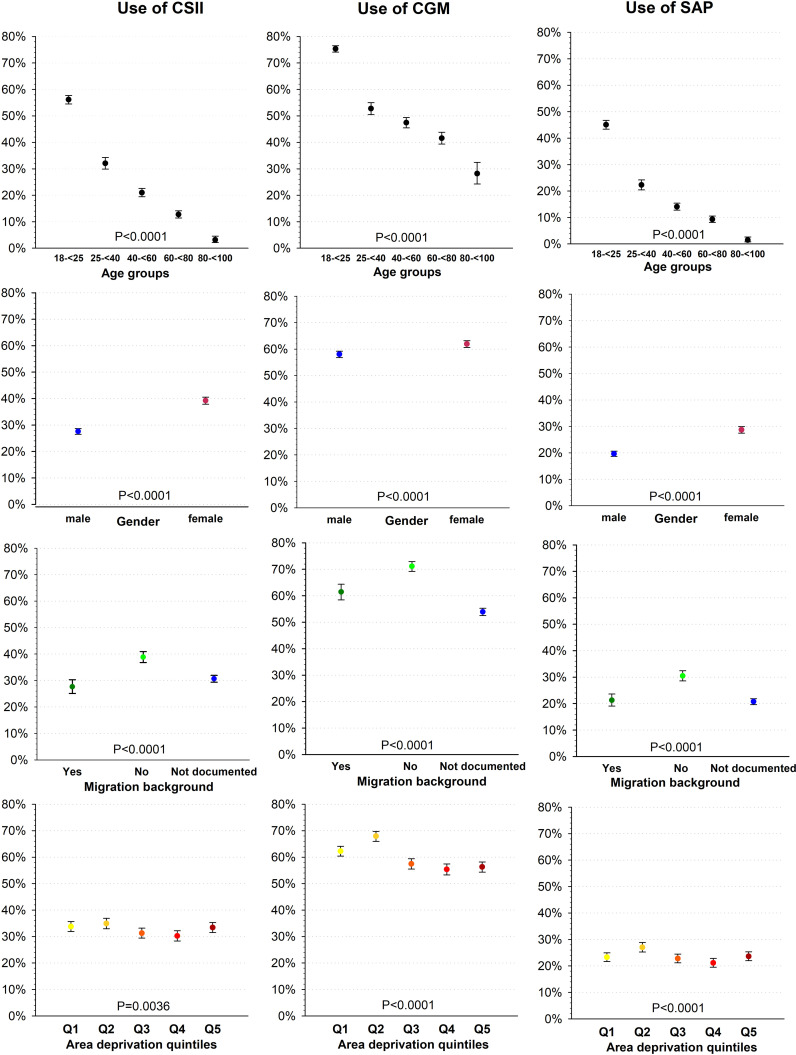
Use of diabetes technology by age group, gender, migration background, and area deprivation.

The use of every technology decreased continuously and significantly with older age (p for trend <0.001, **Figure 1** and **Table 2**). The biggest relative difference in use between two successive age groups was for all devices between the two youngest and between the two oldest age groups (18-<25 vs. 25-<40-year-olds and 60-<80 vs. ≥ 80-year-olds, **Figure 1**). Between the two youngest age groups, CSII use decreased from 56.1% [95%-CI: 54.5−57.7] to 32.1% [29.9−34.3], CGM use from 75.3% [74.1−76.5] to 52.8% [50.5−55.0], and SAP use from 45.1% [43.4−46.7] to 22.3% [20.5−24.2], all differences p <0.001. Between the two oldest age groups, CSII use decreased from 12.7% [11.4−14.1] to 3.1% [2.1−4.6], CGM use from 41.6% [39.3−43.9] to 28.2% [24.3−32.5], and SAP use from 9.3% [8.2−10.5] to 1.5% [0.1−2.7], all differences p <0.001.

**Table 2:** Technology use: coefficients from multiple logistic regression models.

		Use of CSII	P-value	Use of CGM	P-value	Use of SAP (without AID)	P-value	Use of AID	P-value
**Intercept**		- 2.38	<0.0001	- 0.93	<0.0001	- 3.43	<0.0001	- 17.23	<0.0001
**Diabetes duration groups**	**< 5 yrs**	- 2.09	<0.0001	- 0.13	0.0129	- 1.62	<0.0001	- 0.97	<0.0001
	**5-< 10 yrs**	- 1.15		- 0.10		- 0.78		- 0.52	
	**10-< 20 yrs**	- 0.60		- 0.17		- 0.42		- 0.42	
	**≥ 20 yrs**	Ref.		Ref.		Ref.		Ref.	
**Age groups**	**18 -< 25 yrs**	3.50	<0.0001	1.58	<0.0001	3.44	<0.0001	14.96	<0.0001
	**25 -< 40 yrs**	2.67		1.00		2.77		14.14	
	**40 -< 60 yrs**	2.12		0.85		2.26		13.72	
	**60 -< 80 yrs**	1.51		0.61		1.79		13.42	
	**80 -< 100 yrs**	Ref.		Ref.		Ref.		Ref.	
**Gender**	**male**	- 0.53	<0.0001	- 0.16	<0.0001	- 0.48	<0.0001	- 0.27	0.0015
	**female**	Ref.		Ref.		Ref.		Ref.	
**Migration background**	**yes**	- 0.13	<0.0001	0.34	<0.0001	- 0.04	<0.0001	0.39	<0.0001
	**no**	0.37		0.76		0.35		0.79	
	**n.d.**	Ref.		Ref.		Ref.		Ref.	
**Area deprivation quintiles**	**Q1**	0.02	0.0028	0.25	<0.0001	0.08	<0.0001	- 0.46	0.0124
	**Q2**	0.06		0.49		0.20		- 0.07	
	**Q3**	- 0.11		0.00		- 0.08		- 0.02	
	**Q4**	- 0.15		- 0.04		- 0.14		- 0.12	
	**Q5**	Ref.		Ref.		Ref.		Ref.	

Coefficients from logistic regression models, adjusted by diabetes duration, and when possible by age groups, gender, migration background and area deprivation. n.d., not documented.

### 3.2 Technology use by gender

All devices were more frequently used by women than by men (all differences p <0.001, **Figure 1** and **Table 2**). The largest difference between genders was for CSII: 39.2% [37.9−40.6] in women vs. 27.6% [26.5−28.7] in men. CGM was used by 61.9% [60.6−63.2] of the women compared to 58.0% [56.8−59.2] of the men, and SAP by 28.7% [27.5−30.0] of the women compared to 19.6% [18.7−20.7] of the men.

### 3.3 Technology use by migration background

Information on migration background was only documented in 5,290 of 13,351 (39.6%) individuals (**Table 1**). In patients with this information, the use of every technology was significantly higher in individuals without migration background than in those with migration background (all differences p <0.001, **Figure 1** and **Table 2**): CSII was used by 38.8% [36.8−40.9] vs. 27.6% [25.1−30.3], CGM by 71.1% [69.2−72.9] vs. 61.4% [58.5−64.4], SAP by 30.5% [28.6−32.4] vs. 21.3% [19.1−23.6]. In individuals with unknown migration status, CSII was used by 30.6% [29.4−32.0], CGM by 53.9% [52.5−55.3], and SAP by 20.7% [19.6−21.9].

### 3.4 Technology use by area deprivation

Associations between area deprivation and technology use were not linear (**Figure 1** and **Table 2**). The use of every technology decreased with higher deprivation from Q2 to Q4. CGM use was also higher in the two least deprived quintiles Q1-Q2 than in the three most deprived quintiles (Q3-Q5): 62.3%−67.9% vs. 55.4%−57.5%.

### 3.5 Technology use by interaction between migration background and area deprivation

For each type of technology, results from multiple regression models including all factors together (gender, age group, migration background, and area deprivation) were very similar to the results presented above and all factors remained significant (p <0.01).

Interactions between migration background and area deprivation were not significant (CSII: p= 0.794; CGM: p= 0.201; CSII: p= 0.782). The use of each technology was constantly higher in patients without migration background than in those with migration background regardless of deprivation quintile. In patients without migration background, the use of CSII varied in a nonlinear manner across deprivation quintiles between 41.7% (Q1) and 53.1% (Q3), the use of CGM between 76.6% (Q2) and 79.2% (Q3), and the use of SAP between 35.6% (Q1) and 44.3% (Q3). In patients with migration background, the use of insulin pump varied between 32.0% (Q1) and 41.2% (Q3), the use of CGM between 67.4% (Q5) and 75.0% (Q1), and the use of SAP between 26.4% (Q1) and 33.6% (Q3).

### 3.6 HbA1c by technology use

Adults using CSII, CGM or SAP had lower HbA1c in each gender, age, migration, and deprivation category than adults not using these devices (**Table 3**). All comparisons were significant, excepted in adults aged 80 or over (due to their small number, n=468), and in persons without migration background or in persons living in districts Q2 for the use of CSII (**Table 3**).

**Table 3:** HbA1c: results from multiple linear regression models.

		Use of CSII		P-value	Use of CGM		P-value	Use of SAP		P-value
		no	yes		no	yes		no	yes	
**Age groups**	**18 -< 25 yrs**	8.18 [8.12−8.24]	7.94 [7.88−8.01]	<0.0001	8.39 [8.30−8.48]	7.96 [7.91− 8.01]	<0.0001	8.18 [8.13− 8.24]	7.90 [7.83 −7.97]	<0.0001
	**25 -< 40 yrs**	8.34 [8.23−8.45]	7.65 [7.50−7.80]	<0.0001	8.57 [8.45−8.69]	7.70 [7.59− 7.81]	<0.0001	8.28 [8.18 −8.38]	7.56 [7.38−7.73]	<0.0001
	**40 -< 60 yrs**	8.05 [7.98− 8.13]	7.65 [7.53−7.77]	<0.0001	8.24 [8.15−8.33]	7.63 [7.55−7.72]	<0.0001	8.02 [7.95−8.09]	7.60 [7.45−7.75]	<0.0001
	**60 -< 80 yrs**	7.73 [7.67−7.80]	7.42 [7.29−7.55]	<0.0001	7.85 [7.77−7.93]	7.45 [7.36 −7.53]	<0.0001	7.73 [7.66−7.79]	7.32 [7.17−7.48]	<0.0001
	**80 -< 100 yrs**	7.98 [7.84−8.11]	7.41 [6.84−7.99]	0.0611	8.01 [7.86− 8.17]	7.80 [7.56− 8.03]	0.1366	7.96 [7.83 −8.09]	7.42 [6.59 −8.25]	0.2095
**gender**	**male**	8.03 [7.98−8.08]	7.80 [7.73−7.88]	<0.0001	8.29 [8.23− 8.36]	7.73 [7.68−7.78]	<0.0001	8.03 [7.98− 8.08]	7.74 [7.65−7.82]	<0.0001
	**female**	8.18 [8.12− 8.24]	7.73 [7.67−7.80]	<0.0001	8.29 [8.22− 8.36]	7.81 [7.75− 7.86]	<0.0001	8.14 [8.09 −8.19]	7.69 [7.62−7.77]	<0.0001
**Migration background**	**yes**	8.46 [8.33− 8.59]	8.02 [7.85−8.19]	<0.0001	8.71 [8.53− 8.88]	8.11 7.99− 8.23]	<0.0001	8.45 [8.33− 8.57]	7.97 [7.78 −8.15]	<0.0001
	**No**	8.02 [7.95−8.10]	7.92 [7.85−7.99]	0.0609	8.21 [8.10− 8.32]	7.91 [7.85−7.96]	<0.0001	8.04 [7.97 −8.10]	7.88 [7.80− 7.96]	0.0054
	**n.d.**	8.06 [8.02− 8.10]	7.60 [7.52− 7.67]	<0.0001	8.22 [8.16− 8.27]	7.64 [7.59− 7.69]	<0.0001	8.03 [7.99−8.07]	7.52 [7.43−7.60]	<0.0001
**Area deprivation quintiles**	**Q1**	7.82 [7.75−7.90]	7.56 [7.46−7.66]	<0.0001	7.96 [7.87−8.05]	7.58 [7.51−7.65]	<0.0001	7.81 [7.74−7.88]	7.50 [7.39− 7.61]	<0.0001
	**Q2**	7.92 [7.84− 8.00]	7.77 [7.67− 7.87]	0.0265	8.16 [8.05−8.27]	7.73 [7.65−7.80]	<0.0001	7.93 [7.85− 8.00]	7.72 [7.61−7.83]	0.0033
	**Q3**	8.11 [8.02− 8.19]	7.71 [7.59−7.82]	<0.0001	8.37 [8.26−8.47]	7.68 [7.60−7.77]	<0.0001	8.09 [8.01−8.17]	7.63 [7.50−7.76]	<0.0001
	**Q4**	8.38 [8.29−8.47]	7.97 [7.83−8.10]	<0.0001	8.57 [8.45− 8.68]	7.99 [7.89− 8.09	<0.0001	8.34 [8.26−8.43]	7.94 [7.79 −8.09]	<0.0001
	**Q5**	8.28 [8.20− 8.36]	7.84 [7.73−7.95]	<0.0001	8.40 [8.30−8.49]	7.90 [7.81− 7.99]	<0.0001	8.25 [8.18 −8.33]	7.77 [7.64−7.89]	<0.0001

Linear regression models adjusted by diabetes duration, when possible by gender and by age groups. n.d., not documented.

## 4 Discussion

Our analysis based on more than 13,000 adults with T1D in Germany provides real world evidence that younger age, female gender, and absence of migration background are significant facilitators for use of diabetes technology in this population. Associations with area deprivation were less clear.

Previous real-world analyses from Germany reported a higher use of diabetes technology with younger age in pediatrics, as well as an overall lower use in adults compared to children (1, 27). However, the impact of age on the use of diabetes technology within the adult population has not been investigated to date. German and international guidelines recommend the use of diabetes technology (CSII, CGM, and also AID) for most adults, even older ones, if they desire it and if this use is compatible with preserving their autonomy (28, 29). Yet, our data indicate that the real-world use of CSII, CGM and SAP significantly decreases with older age. Data from France also confirmed a lower use of CSII with older age in adults (30). In contrast, data from the US-T1D Exchange registry indicated the lowest use of both CGM and CSII in 18-25 year-olds compared to older patients (3, 31). The high cost and lack of reimbursement for these technologies in the absence of health insurance may explain the lower use of these technologies by young adults in the US, since young adults tend to have lower incomes than their elders. In Germany, nearly all patients benefit from a health insurance. Moreover, the higher initiation rate in children and adolescents in this country and the continuation of technology use after childhood may contribute to the higher use in young adults. Barriers related to difficulties with technology utilization seem not to play a role for age differences, except perhaps in the oldest age group, in which disabilities may limit the use of these devices (23, 31). Nevertheless, the impact of age on technology use in adults needs to be further investigated.

We found a higher use of all technologies in women compared to men, with the largest difference for CSII. To date, numerous studies reported a higher use of CSII or SAP, but not of CGM alone, in female adolescents and adults (1, 4, 27, 30–34). This finding is consistent in many reports, although women often report more physical barriers to technology adoption than men (23, 31). Several specific indications for technology use for women exist. Current German guidelines recommend for instance the use of CSII and of CGM for women before and during pregnancy (28, 35). CSII is also indicated in case of unsatisfying glycemic control, which is more frequent in female adolescents compared to males (33). The more frequent use of a pump in young women may continue with older age even if the glycemic results improve (33). In contrast to older studies, our data indicate that women used a CGM more frequently than men. The greater use of SAP and AID in women compared to men in the most recent years leads automatically to a higher CGM use, since a CGM is part of all SAP and AID systems.

To date, only few studies have examined demographic and socioeconomic disparities in technology access in adults with T1D (16, 17, 31, 36). An analysis from the UK indicates an association between higher deprivation and lower use of CSII and CGM in adults with T1D, as well as a significant lower use of both technologies in individuals with black ethnicity compared to those with mixed or white ethnicity (18). In our analysis, differences in technology use by migration background were stronger than those by area deprivation and the use of each technology was constantly higher in adults without migration background regardless of deprivation. These results are consistent with previous findings in pediatrics in Germany (15). Contrary to what is known about the situation in England (18) or the United States (20, 37, 38), there is no strong correlation between migration background and regional deprivation in Germany, because less migrants live in the most deprived areas (e.g. in eastern parts of Germany) than in the least deprived areas (e.g. in Bavaria and in Baden-Württemberg) (39, 40). In our study population with documented migration status, the highest proportion of persons with migration background lived in moderately deprived area (Q3: 27.6% vs. 21.9-24.8% in the other districts). In addition, almost all adults living in Germany have a statutory or private insurance that reimburses most of CSII and CGM costs in case of intensive insulin therapy. Thus, in contrast to the situation in the US where individuals might be disadvantaged due to their insurance status (31, 38, 41), economic factors should not play an important role in limiting access to technologies for T1D in Germany.

We found, however, that the presence of migration background was significantly associated with less technology use. Individuals with migration background have less often a higher qualification degree than German natives (42) and some first generation migrants may have difficulties with the language of the host country. This can constitute a barrier to complete the specialized education required to use diabetes treatment devices (31). Initial and ongoing education and training is essential for the use of diabetes technology, but it requires a number of resources, like free time, health literacy and numeracy or perceived self-efficacy (6, 43). Language barriers may also exist when it comes to filling out forms for reimbursement or telephone contact when technical problems with diabetes devices arise (31). Finally, the choice of a specific device must be based on individual characteristics, that is a person’s needs, preferences and skills levels (6). In this decision-making process, the subjectivity of both the patient and the provider play a role. As a consequence, provider implicit bias, observed for example when the recommendation of diabetes technology unconsciously but systematically disadvantages some patients due to their ethnic or socioeconomic characteristics, is always possible and may also exist in Germany (38, 42, 44).

Our results indicate better glycemic control in all adults using CSII, CGM or SAP compared to those not using these technologies. This is an argument for continuing efforts to improve access to technologies in older adults, in males and in people with migration background. However, due to the cross-sectional design of this study, these associations must be interpreted with caution and we cannot conclude on a potential causal relationship between technology use and lower HbA1c.

### 4.1 Strengths and limitations

One strength of this study is the use of the large multicenter DPV registry, which can give a good insight into the real-world use of diabetes technology in adults with T1D in Germany. Even if the representativeness of the registry is lower than in pediatric diabetes, the risk of selection bias in our findings is relatively low and generalizations may be valid. However, given the rapid advances in diabetes technology and the continued increase in its use, these analyses must be updated regularly. One limitation is that socioeconomic factors were assessed at the district-level, not at the individual level. Aggregated data can weaken the effect of individual socioeconomic factors on the use of diabetes technology and underestimate their influence. Nevertheless, other aspects related to living conditions and diabetes care, which is largely organized at the federal level, can be better reflected using an area-based deprivation index. We did not account for persons who moved from one district to another and thus potentially changed their deprivation category. However, only 4.6% of the population have moved within Germany in 2021 (destatis.de) and only a part of this proportion may have moved to a different deprivation quintile. Moreover, some of them might have moved to a more deprived district, but others to a less deprived district, so that the resulting potential bias may be mainly non-differential. Finally, we used a binary variable for migration background that does not reflect the tremendous heterogeneity within the population. In 2021, more than a quarter of the people living in Germany had a migration background (45). These persons form a very heterogeneous subpopulation in terms of country of origin, time living in Germany, reasons for migration, legal status, education, language skills, or access to employment. Our results do not take this diversity into account and this could be the subject of future research.

## 5 Conclusion

Our real-world data provide evidence that higher age, male gender, and migration background are associated with lower use of modern diabetes technology in adults with T1D in Germany. Associations with area deprivation are more complex, probably due to correlations with other factors that exert in part opposite effects, like the higher proportion of migrants in less deprived areas, or the federal structure of the German health care system. There is a critical need to improve access to diabetes technology in underserved groups for reducing health disparities. This can enable them to benefit from the latest technological advancements and achieve better glycemic control, which has the potential to ultimately lead to improved health outcomes.

## Data availability statement

Access to the programming code can be provided by the corresponding author upon request. For reasons of data protection, data on individual level cannot be provided. However, remote data analysis is possible. Requests to access these datasets should be directed to marie.auzanneau@uni-ulm.de.

## Author contributions

MA and RWH designed the study. MA and AJE analyzed the study data. MA created the figures and wrote the manuscript. AE, SMM, MHe, AG, LS, PMJ, MHu, and RWH contributed to the discussion, reviewed, and approved the manuscript. All authors contributed to the article and approved the submitted version.
